# Positron Emission Tomography Imaging of Endometrial Cancer Using Engineered Anti-EMP2 Antibody Fragments

**DOI:** 10.1007/s11307-012-0558-y

**Published:** 2012-05-15

**Authors:** Maoyong Fu, Sarah Brewer, Tove Olafsen, Anna M. Wu, Lynn K. Gordon, Jonathan Said, Jonathan Braun, Madhuri Wadehra

**Affiliations:** 1 Department of Pathology and Laboratory Medicine, David Geffen School of Medicine at UCLA, Los Angeles, CA 90095 USA; 2 Department of Molecular and Medical Pharmacology, David Geffen School of Medicine at UCLA, Los Angeles, CA 90095 USA; 3 Jonsson Comprehensive Cancer Center, David Geffen School of Medicine at UCLA, Los Angeles, CA 90095 USA; 4 Department of Ophthalmology, Jules Stein Eye Institute, David Geffen School of Medicine at UCLA, Los Angeles, CA 90095 USA; 5 Department of Surgery, Greater Los Angeles Veterans Affairs Healthcare System, Los Angeles, CA 90099 USA

**Keywords:** Epithelial membrane protein-2, Endometrial cancer, PET imaging, Minibody, Copper-64

## Abstract

**Purpose:**

As imaging of the cell surface tetraspan protein epithelial membrane protein-2 (EMP2) expression in malignant tumors may provide important prognostic and predictive diagnostic information, the goal of this study is to determine if antibody fragments to EMP2 may be useful for imaging EMP2 positive tumors.

**Procedures:**

The normal tissue distribution of EMP2 protein expression was evaluated by immunohistochemistry and found to be discretely expressed in both mouse and human tissues. To detect EMP2 in tumors, a recombinant human anti-EMP2 minibody (scFv-hinge-C_H_3 dimer; 80 kDa) was designed to recognize a common epitope in mice and humans and characterized. In human tumor cell lines, the antibody binding induced EMP2 internalization and degradation, prompting the need for a residualizing imaging strategy. Following conjugation to DOTA (1,4,7,10-tetraazacyclododecane-*N*,*N*′,*N*′,*N*′″-tetraacetic acid), the minibody was radiolabeled with ^64^Cu (*t*_1/2_ = 12.7 h) and evaluated in mice as a positron emission tomography (PET) imaging agent for human EMP2-expressing endometrial tumor xenografts.

**Results:**

The residualizing agent, ^64^Cu-DOTA anti-EMP2 minibody, achieved high uptake in endometrial cancer xenografts overexpressing EMP2 (10.2 ± 2.6, percent injected dose per gram (%ID/g) ± SD) with moderate uptake in wild-type HEC1A tumors (6.0 ± 0.1). In both cases, precise tumor delineation was observed from the PET images. In contrast, low uptake was observed with anti-EMP2 minibodies in EMP2-negative tumors (1.9 ± 0.5).

**Conclusions:**

This new immune-PET agent may be useful for preclinical assessment of anti-EMP2 targeting *in vivo*. It may also have value for imaging of tumor localization and therapeutic response in patients with EMP2-positive malignancies.

## Introduction

A promising avenue in molecular imaging is the development of agents that detect the presence and levels of molecular biomarkers in malignant tumors. In this way, molecular imaging may provide important information for patient stratification to targeted therapies, aid in restaging diseases, and be used to monitor treatment response. Thus, new biomarkers that may aid in patient management are urgently needed.

Epithelial membrane protein-2 (EMP2) is a novel oncogene upregulated in a number of gynecological tumors. A member of the growth arrest specifϊc-3/peripheral myelin protein-22 (GAS3/PMP22) family of tetraspan proteins, overexpression of EMP2 is associated with tumor progression as well as poor patient survival [1, 2]. Its expression is detected in up to 60 % of endometrial tumors and 75 % of advanced ovarian tumors [1–3].

Functionally, EMP2 appears to be important for trafficking, intracellular compartmentalization, and the surface display of selected receptors and glycolipids [4]. EMP2 physically associates with and regulates the activity of integrin-FAK signaling complexes, with the consequence that high levels of EMP2 increase tumor cell invasion [5–8]. These findings, concordant with the role of other members of the tetraspan family, indicate that EMP2 organizes molecules at the cell surface that are brought into engagement with specific signaling complexes, therefore controlling downstream biological responses [9–12].

We have previously reported an anti-EMP2 diabody (scFv dimer; 55 kDa) derived from a human immunoglobulin V-gene phage display library that recognizes a shared epitope on murine and human EMP2 [13]. Localized treatment with the anti-EMP2 diabody showed therapeutic efficacy with reduced tumor load in xenograft models for endometrial and ovarian tumors. Given that EMP2 overexpression plays an important role in aggressive tumor behavior and poor clinical outcome [1], early stage detection and quantification of EMP2 may be clinically relevant and used for selection of optimal therapy for individual patients.

Positron emission tomography (PET) imaging is a widely used imaging modality due to its higher image resolution and sensitivity compared to single-photon imaging [14]. In oncology, PET most commonly employs 2-deoxy-2-[^18^F]fluoro-d-glucose ([^18^F]FDG) as a tracer for imaging elevated metabolism in tumors [15]. However, [^18^F]-FDG has certain limitations in tumor detection. First, it is not specific for tumor detection, as inflammation and wound healing also exhibit tissue uptake of [^18^F]-FDG [16]. Second, with regard to gynecological tumors, [^18^F]-FDG uptake is deficient in a substantial fraction of gynecologic malignancies and can be insensitive for assessing responses to certain antitumor therapies [17–19].

Here, we have generated a larger anti-EMP2 antibody fragment, a minibody (scFv-hinge-C_H_3 dimer; 80 kDa). In order to determine if EMP2 could be detected *in vivo*, the minibody was conjugated to DOTA and radiometal labeled with the positron emitter ^64^Cu (T_1/2_ = 12.7 h). Small animal PET imaging showed excellent tumor targeting of the ^64^Cu-DOTA-minibody in mice bearing EMP2 positive endometrial tumor xenografts. Thus, whole-body PET detection of this anti-EMP2 antibody fragment may have promise as a potential imaging agent for staging and monitoring of EMP2-positive endometrial tumors.

## Materials and Methods

### Cell Lines and Cell Culture

HEC1A/EMP2 cells were generated by stable transfection with the EGFP-N3 expression vector bearing human EMP2 as previously described [7]. Ramos cells were purchased from American Type Culture Collection (ATCC, Manassas, VA, USA), and the murine mammary tumor D2F2 was kindly provided by Dr. Manuel Penichet (UCLA). The cells were cultivated at 37 °C in a humidified 5 % CO_2_ in DMEM medium (Mediatech, Manassas, VA, USA) supplemented with 10 % fetal calf serum (Hyclone Laboratories, Logan, UT, USA), 2 mM l-glutamine, 1 mM sodium pyruvate, 100 U/ml penicillin, and 100 U/ml streptomycin (all from Invitrogen Life Technologies, Carlsbad, CA, USA).

### Institutional Review

All procedures with and tissue procurement from mice were performed under Institutional Review Board (IRB) approval. All human samples were obtained from the UCLA Department of Pathology and Laboratory Medicine under Institutional Review Board exemption approval.

### Construction and Production of Anti-EMP2 Antibody Fragments

The anti-EMP2 minibody was generated using published methods [20]. Briefly, genes encoding the variable KS83 domain were assembled by PCR into a single-chain Fv fragments (scFv) [13] and cloned into pCR2.1-TOPO vector (Invitrogen). The genes encoding the human IgG1 hinge and C_H_3 domain (hinge-C_H_3) were also assembled by PCR and cloned into another pCR2.1-TOPO vector. The pCR2.1-TOPO-scFv and scFv-hinge-C_H_3 were digested and inserted into the pcDNA3.1myc/his(−) mammalian expression vector (Invitrogen) at *Xba*I and *EcoR*I site and *Xho*I and *EcoR*I, respectively. The resultant anti-EMP2 minibody was termed KS83.

Chinese hamster cells (CHO-K1) were transfected with 30 μg of linearized pcDNA3.1-KS83 vector DNA and selected using 1 mg/ml of geneticin (G418, Sigma-Aldrich, St. Louis, MO, USA) as described previously [21]. After approximately 2 weeks, cells were screened for expression by enzyme-linked immunosorbent assay (ELISA) using goat anti-human Fc-specific antibody (Jackson Immunoresearch, West Grove, PA, USA). The highest producers were identified, and the cells were subcloned through serial dilutions to isolate individual clones. Cells from high expressing individual clones were expanded in Celline AD 1000 bioreactor flasks (IBS Integra Biosciences, Hudson, NH, USA) according to the manufacturer’s instructions. The KS83 minibody was purified from supernatant by Protein-L affinity chromatography (Thermo Fisher Scientific Inc., Rockford, IL, USA), using phosphate-buffered saline (PBS) as running buffer. The elution buffer was using 30 % 0.2 M citrate buffer (pH 2.1) in PBS into 80 % *v*/*v* 1 M Tris base, pH 8.2. Purified minibody was dialyzed against PBS and concentrated using Amicon Ultra-4 (Millipore, Billerica, MA, USA). The final concentration of purified minibodies was determined by a Nanodrop 2000 (Thermo Scientific).

### Enzyme-linked immunosorbent assay

The anti-EMP2 antibody fragments were captured by the peptides corresponding to the extracellular loop of human EMP2 [13], and ELISA was performed as described previously [13]. Specifically, bound minibodies were detected with horseradish peroxidase (HRP)-conjugated goat antihuman Fc-specific antibody (Jackson Immunoresearch), followed by tetramethylbenzidine solution (eBioscience, San Diego, CA, USA). Plates were read using a Model 550 microplate reader (Bio-Rad, Hercules, CA, USA) at 450 nm.

### Flow Cytometry

HEC1A/EMP2 cells (1 × 10^6^), murine D2F2 cells, or Ramos cells suspended in 1 ml of flow buffer (PBS, 0.2 % bovine serum albumin, and 0.02 % sodium azide), were centrifuged for 5 min at 500×*g*, 4 °C. Cells were resuspended in 0.1 ml flow buffer and incubated with 0.5 μg of the primary antibody for 1 h at 4 °C on a rotator. Cells were washed three times with flow buffer and incubated for 30 min at 4 °C with phycoerythrin (PE)-conjugated goat antihuman Fc-specific antibody (Jackson Immunoresearch) in 0.1 ml flow buffer. Following three washes, cells were resuspended in 0.4 ml flow buffer, and flow cytometry was immediately performed. Fc receptors on Ramos cells were blocked by incubation for 15 min with 50 μl Fc block solution (heat inactivated rabbit normal serum, 1:10 dilute in PBS, freeze–thaw five cycles).

### Immunohistochemistry

Mouse and human tissues were stained for EMP2 expression as previously described [22]. Normal human tissue were stained using an array created by the immunohistochemistry core of the Translational Pathology Core Laboratory. This array contains 248 spots from 30 tissues with examples of every type from at least two patients, and all samples were verified as being normal by histology. All positive staining was verified independently using additional full tissue sections from two additional patients. For mouse tissue, organs were extracted, tissue fixed in 10 % formalin, embedded in paraffin, and sectioned at 5-μm thickness. To assess EMP2 expression in all tissue, antigen retrieval was performed using 0.1 M citrate buffer (pH 6.0) at 95 °C for 20 min. The slides were incubated with rabbit antimouse EMP2 antisera (1:250) [23], antihuman EMP2 antisera (1:800) [1] or control rabbit serum at the same dilutions overnight. The antibody signal was visualized using the Vectastain ABC kit (Vector Labs, Burlingame, CA, USA) according to the manufacturer’s instructions. EMP2 expression was detected using diaminobenzidine, and nuclei were counterstained using hematoxylin. Semiquantitative scoring of the EMP2-stained tissue was conducted using a 0–3 intensity scale.

### Western Blot Analysis

Mouse tissues were processed using fresh specimens and immediately frozen on dry ice. Frozen human tissue samples of postdiagnostic remnant surgical resection specimens obtained from the UCLA Department of Pathology and Laboratory Medicine. Tissues were homogenized using cold radioimmunoprecipitation assay buffer and then resuspended in Laemmli buffer. To detect EMP2 expression, cell extracts were treated with peptide N-glycosidase F (New England Biolabs, Beverly, MA, USA) to deglycosylate the proteins. The lysates were separated on a 12 % sodium dodecyl sulfate polyacrylamide gel electrophoresis (SDS-PAGE) gel, and proteins were transferred onto nitrocellulose (Bio-Rad Laboratories, Hercules, CA, USA). EMP2 was detected using rabbit antihuman EMP2 (1:2000) antiserum followed by a HRP-conjugated goat antirabbit IgG and enhanced chemiluminescence detection reagents (GE Healthcare, Piscataway, NJ, USA).

### Immunofluorescence

HEC1A/EMP2 cells were plated onto glass coverslips (Fisher Scientific, Pittsburgh, PA, USA). Cells were washed in PBS and then incubated at various time points with 20 μg/ml of the control A10 or the anti-EMP2 diabody. To visualize the diabody, cells were fixed in 1.6 % formaldehyde for 20 min followed by incubation with a fluorescein isothiocyanate-conjugated anti-Myc antibody (Invitrogen). To visualize EMP2 staining, cells were fixed and permeabilized in methanol at −20 °C for 30 min and rehydrated in PBS. Cells were blocked in 1 % normal goat serum for 45 min and incubated overnight at 4 °C with rabbit antihuman EMP2 antisera (1:400) in a humidified chamber. Cells were rinsed with PBS/0.01 % Triton X-100 and then incubated (2–4 h at room temperature) with rhodamine-conjugated goat antirabbit IgG (1:4,000; Jackson Immunoresearch). Negative controls included incubation of cells with secondary antibody alone. Cells were copiously washed in PBS/0.01 % Triton X-100, rinsed briefly with double deionized H_2_O, and mounted in Vectamount HardSet with 4′,6-diamidino-2-phenylindole solution. Images were taken using an Olympus BX51 light microscope coupled with the DP72 digital camera at 400× magnification.

### DOTA Conjugation and Radiolabeling with ^64^Cu

The KS83 minibody was conjugated with 1,4,7,10-tetraazacyclododecane*N*,*N*′,*N*″,*N*′″-tetraacetic acid mono-*N*-hydroxysuccinimide ester (DOTA-NHS-ester; Macrocyclics, Dallas, TX, USA) according to the method described previously [24]. Different conjugation molar ratios of DOTA to minibody (20, 50, 100, and 1,000) were used in order to determine the condition that would preserve the binding activity of the conjugate. ^64^Cu (copper chloride in 0.1 M HCl; radionuclide purity, >99 %) was provided by the Mallinckrodt Institute of Radiology (Washington University School of Medicine). For radiometal labeling, 200 μg of DOTA-conjugated KS83 was incubated with 400 μCi (14.8 MBq) of ^64^Cu in 0.1 M NH_4_ citrate (pH 5.5) for 50 min at 43 °C. The radiolabeling efficiency was determined using instant thin-layer chromatography with Tec-Control Chromatography strips according to the manufacturer’s instruction (Biodex Medical Systems, Shirley, NY, USA). In some experiments, the anti-CD20 minibody was used as a negative control isotype tracer [25]. DOTA conjugation and radiolabeling with ^64^Cu were performed in the same way as above.

### Immunoreactivity of Radiolabeled Minibody

Immunoreactivity was measured as previously described [21]. In this study, HEC1A/EMP2 cells (6–9 × 10^7^) in excess were incubated with 20 ng of ^64^Cu-DOTA-KS83 for 1 h on ice. Cells were centrifuged at 1,000×*g* for 5 min, and the activity remaining in the supernatant was counted using a Wallac WIZARD automatic γ-counter (Perkin-Elmer Life and Analytic Sciences Inc., Waltham, MA, USA). The immunoreactivity (%IR) of the radiolabeled antibody was calculated with the following formula: $\% {\text{IR}} = {1}00 - \left( {{\text{activity}}\,{\text{from}}\,{\text{the}}\,{\text{tubes}}\,{\text{with}}\,{\text{cells}}/{\text{activity}}\,{\text{from}}\,{\text{control}}\,{\text{tubes}}\,{\text{without}}\,{\text{cells}}\, \times {1}00} \right)$.

### Tumor Xenograft Murine Model

All procedures involving animals were performed under approved protocols of the UCLA Animal Research Committee. Six to 8-week-old female BALB/c nude mice (Charles River Labs, Wilmington, MA, USA) were injected subcutaneously in the shoulder with 1 × 10^5^ of HEC1A/EMP2 or wild-type HEC1A cells 3–4 weeks prior to imaging. 5 × 10^6^ Ramos cells were injected to form the subcutaneous tumor 1–2 weeks prior to imaging. Each group utilized five mice per experiment.

### Small-Animal PET Imaging

Mice were anesthetized with 2 % isoflurane prior to intravenous injection of ~100 μCi (3.7 MBq) of ^64^Cu-DOTA-KS83 minibody (50 μg) or ^64^Cu-DOTA-CD20 minibody (50 μg) via tail vein (specific activity, 0.074 MBq/μg). Mice were imaged at 4 and 20 h post-injection. A cylinder of known weight, containing a known amount of radioactivity, was scanned to provide a calibration standard. Mice were serially imaged using the micro-PET Focus 220 PET Scanner (Siemens Preclinical Solutions, Knoxville, TN, USA). To enable imaging, mice were positioned in a prone position along the long axis of the microPET scanner and imaged. Images were reconstructed using a filtered backprojection reconstruction algorithm [26]. Directly after the final microPET scan, a CT scan was performed using a MicroCAT II Scanner (Concorde Microsystems, Knoxville, TN, USA).

### Biodistribution and ROI Analysis

After the final imaging scan, the mice were euthanized, major organs and tumors were removed and weighed, and radioactivity was counted using a Wallac WIZARD automatic γ-counter (Perkin-Elmer Life and Analytic Sciences Inc.). The percent injected dose per gram of tissue (%ID/g) was calculated to represent the radioactivity uptake in tumor and organs and decay corrected.

AMIDE was used to analyze overlaid CT and PET scans [27]. Regions of interest (ROIs) were drawn based on the CT image. ROI statistics were generated using AMIDE and converted to percent injected dose per gram after input of the individual decay corrected dose and cylinder calibration factor.

### Statistical Analysis

All significant differences between groups were evaluated using two-tailed Student’s unpaired *t* test or ANOVA at a 95 % confidence level (GraphPad Prism version 3.0; GraphPad Software, La Jolla, CA, USA), and *P* < 0.05 were considered significant.

## Results

### Expression of the EMP2 Protein

**Fig. 1:**
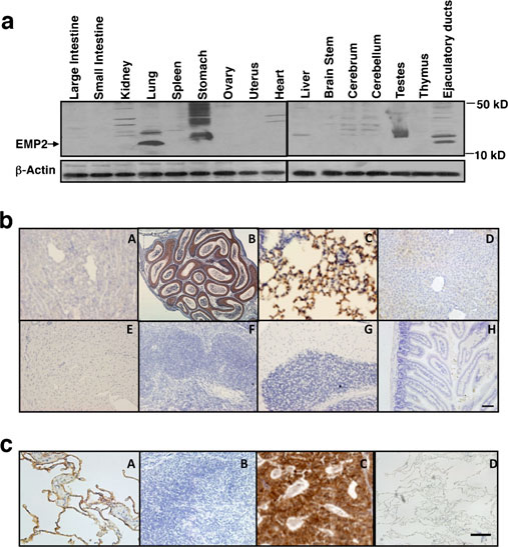
Biodistribution and expression of the EMP2 protein. **a** Mouse tissue lysates were probed for EMP2 expression. The ejaculatory ducts include regions from the epididymis and vas deferens. *Arrow* depicts deglycosylated EMP2 (18 kD). **b** Immunohistochemistry analysis of mouse organs. The organs are designated as follows: *A* kidney, *B* epididymis, *C* lung, *D* liver, *E* heart, *F* spleen, *G* brain, *H* jejunum. **c** Immunohistochemistry analysis of human organs. The representative organs are designated as follows: *A* lung, *B* spleen, *C* endometrial tumor, *D* isotype control in the lung.

To determine the suitability of EMP2 as a new molecular imaging target, its tissue expression in mouse and human was evaluated (Fig. 1). Western blot of mouse tissue lysates revealed high expression of EMP2 in the lung, and some expression in vas deferens (Fig. 1a). Immunohistochemistry confirmed EMP2 expression in the epididymis and lung (panels B and C) and no expression in the kidney, liver, heart, spleen, brain, and jejunum (Fig. 1b, panels A, D, E, F, G, H, respectively). As there is a 76 % amino acid homology between human and mouse EMP2 (GeneBank numbers AAC51779.1 and AAK29076.1), we next evaluated the expression of EMP2 in a human tissue array using anti-EMP2 antisera [22]. Similar to previous reports, EMP2 was highly expressed in alveolar epithelium of the lung [28, 29], in the retinal pigmented epithelium in the eye [22], and within secretory endometrium [23, 30], as well as in the epithelia of the vagina and fallopian tube (Table 1). In contrast, detailed immunohistochemical analysis revealed that EMP2 was undetectable in many human tissues including the small and large intestines, pancreas, liver, spleen, and kidney. To confirm its expression in humans, additional full section tissues were stained. EMP2 expression was readily detectable in the lung (Fig. 1c, panel A) but not in the spleen (panel B). In addition, high levels of EMP2 are seen in representative example of endometrial adenocarcinoma (panel C). The protein expression of EMP2 in these tissues appeared to be specific as no staining was observed using preimmune sera (lung, panel D). The limited normal tissue distribution of human EMP2 makes it an attractive target and suggests its suitability as a molecular target for immunoPET.

**Table 1:** Summary of EMP2 staining in a normal human tissue array

EMP2-positive tissue	EMP2-negative tissue		
Lung	Parathyroid	Thyroid	Testes
Skin	Adipose	Liver	Skeletal muscle
Fallopian tube	Esophagus	Stomach	Small intestine
Placenta	Colon	Anal mucosa	Gall bladder
Vagina	Omentum	Adrenal gland	Pancreas
Uterus	Spleen	Thymus	Parotid gland
	Heart	Kidney	Bladder
	Submandibular gland		Reactive lymph node

### Characterization of Purified KS83 Minibody

**Fig. 2:**
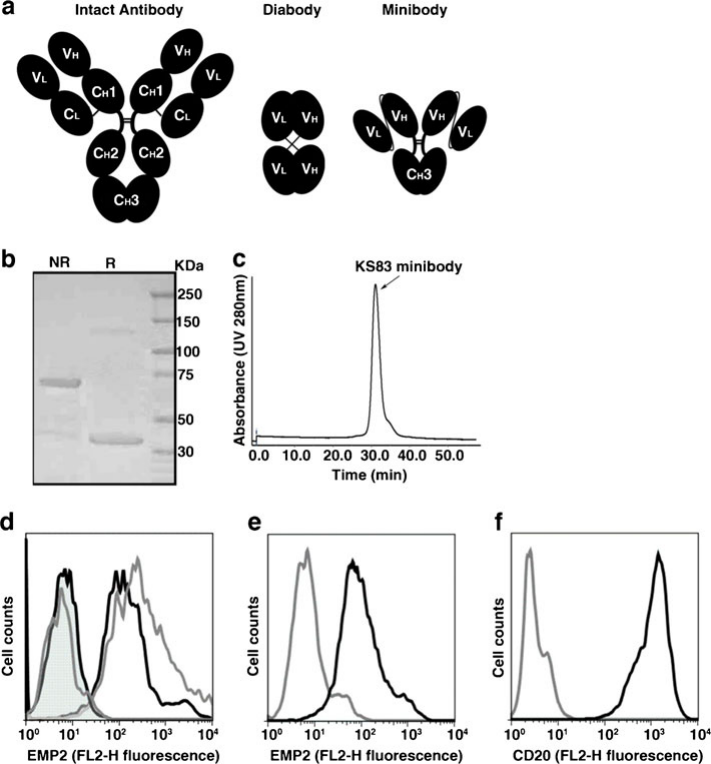
Characterization of purified KS83 minibody. **a** Schematic representation of an intact antibody (150 kDa) and engineered antibody fragments including diabody (scFv dimer, ~55 kDa) and minibody (scFv-hinge-C_H_3 dimer, ~80 kDa). **b** Coomassie blue staining after SDS-PAGE of purified KS83 minibody under non-reducing (NR) and reducing conditions (R). *Lane 3* Molecular weight marker. **c** Size-exclusion chromatography of purified KS83 minibody on a Superdex 200 column. Retention time of the sample was compared with appropriate molecular weight standards. **d** Flow cytometric analysis of HEC1A/EMP2 cells stained with antibody fragments KS83 minibody (*black line*), KS83 diabody (*gray line*), or isotype control anti-CD20 minibody (*tinted black line*) and A10 diabody (*tinted gray line*) and detected by phycoerythrin-conjugated goat antihuman Fc-specific antibody. **e** Flow cytometric analysis of murine D2F2 cells stained with KS83 minibody (*black line*). Anti-CD20 minibody was used as the isotype control (*gray line*) and detected as above. **f** Flow cytometric analysis of Ramos cells stained with anti-CD20 minibody (*black line*). KS83 minibody was used as the isotype control (*gray line*) and detected as above.

Previous experience from our group has shown that the optimum antibody fragment for imaging is an intermediate sized antibody, termed a minibody (Fig. 2a). Minibodies show prolonged serum residence (*T*_1/2β_ = 6 h) compared with a diabody (*T*_1/2β_ = 2–4 h) [31] and have been used successfully in clinical trials to detect tumors prior to surgical excision [32]. The KS83 diabody was modified to create a minibody, and in accord with expectations, purified KS83 minibody formed a covalent homodimer with an estimated molecular mass of 75 kDa under non-reducing conditions (Fig. 2b). Under reducing conditions, the KS83 minibody was dissociated into a monomer of approximately 40 kDa. Size-exclusion chromatography also showed the formation of a dimer with a protein retention time at 31.36 min (average of two experiments), matching with the expected size of the minibody (Fig. 2c [20]). The ability of the KS83 minibody to bind to EMP2 was assessed directly on the HEC1A/EMP2 by flow cytometry. The KS83 minibody demonstrated clear binding to the surface of HEC1A/EMP2 cells, and its affinity was similar to that observed for KS83 diabody (Fig. 2d). In contrast, the isotype control anti-CD20 minibody exhibited no binding to the EMP2-positive cells. To confirm that the KS83 minibody recognized murine EMP2, D2F2 mammary tumor cells were utilized. D2F2 cells have moderate surface expression of EMP2 (Fu and Wadehra, unpublished data), which was detected by the KS83 minibody (Fig. 2e). This effect seemed to be specific as the anti-CD20 minibody showed no binding to D2f2 cells. Furthermore, as shown in Fig. 2f, Ramos cells expressed high levels of CD20, but no EMP2 expression was found on the cells. These results indicate that the KS83 minibody specifically binds to EMP2 and recognizes native EMP2 expressed on the surface of both murine and human cells.

### EMP2 Internalizes Rapidly in the Presence of Anti-EMP2 Fragments

**Fig. 3:**
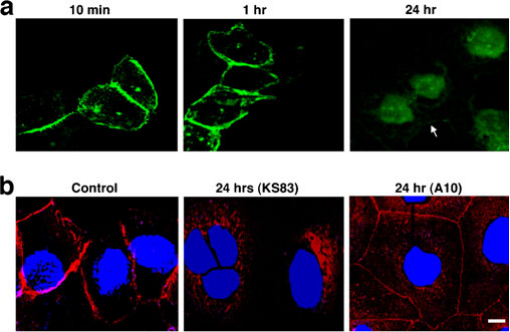
EMP2 internalizes in the presence of anti-EMP2 fragments. **a** Localization of anti-EMP2 antibody (KS83 diabody) fragment (using fluorescein-conjugated anti-Myc antibody) on HEC1A/EMP2 after incubation for 10 min, 1 h, and 24 h. **b** EMP2 localization detected by polyclonal anti-EMP2 antibody (*red*) shows internalization in response 24 h after incubation with anti-EMP2 (KS83 diabody). Isotype control (A10 diabody) antibody fragments or no treatment (control) shows no internalization of EMP2. 4′,6-Diamidino-2-phenylindole was used for nuclear stain (*blue*).

Antibody binding to its receptor often leads to receptor internalization [33, 34]. To determine if EMP2 internalizes upon binding of an anti-EMP2 antibody fragment, light microscopy of HEC1A/EMP2 cells incubated with the anti-EMP2 KS83 diabody was performed using a fluorescein-conjugated anti-Myc antibody. As seen in Fig. 3a, binding of KS83 diabody to HEC1A/EMP2 cells occurred within 10 min and remained cell-associated for up to 1 h, but was lost by 24 h. When the EMP2 levels in response to treatment with the anti-EMP2 antibody fragment was monitored, the EMP2 levels on the HEC1A/EMP2 cell surface were notably reduced within 24 h relative to the negative control cells treated with the A10 isotype control diabody (Fig. 3b). These results suggested that binding of anti-EMP2 antibody fragments leads to receptor internalization and degradation of the antibody fragment.

### DOTA-Conjugated KS83 Minibody and Binding Analysis of Bioconjugates

**Fig. 4:**
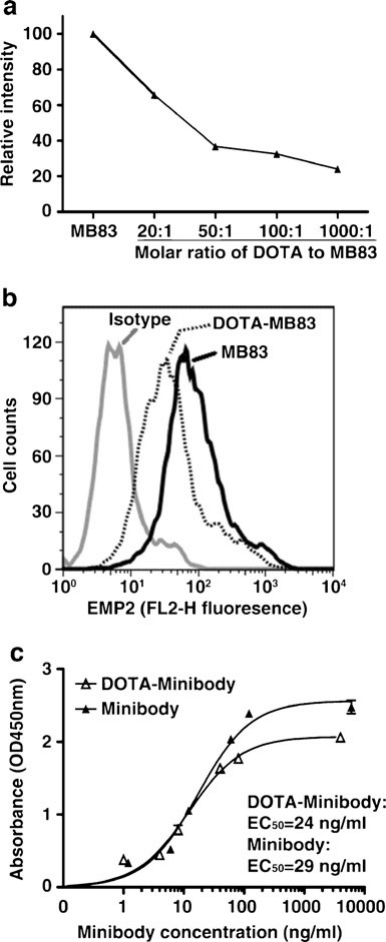
Conjugation of KS83 minibody with DOTA and binding analysis of subsequent bioconjugates. **a** The effect of varying the molar ratios of DOTA to KS83 minibody was determined by flow cytometry using HEC1A/EMP2 cells. The optimal molar ratio of DOTA to KS83 is 20:1, as shown by the best preserved binding activity of the DOTA-KS83 bioconjugate to the cells. **b** Representative flow cytometric image of HEC1A/EMP2 cells stained with unconjugated KS83 minibody and the 20:1 conjugated DOTA-KS83 minibody. Unconjugated KS83 minibody and the isotype control A10 diabody are depicted as the positive and negative controls, respectively. **c** The level of binding of the KS83 and DOTA-KS83 minibody to human EMP2 second extracellular loop peptide was determined by ELISA. Plates were coated with the 24 amino acid human EMP2 peptide, and bound minibodies were detected with HRP-conjugated goat anti-human Fc-specific antibody.

Given the rapid internalization of antibody-bound EMP2, a residualizing radiolabel strategy was employed to visualize tumors *in vivo*. Residualizing radiolabels, such as radioactive metals attached to a bifunctional chelator such as DOTA, become trapped in the cells and are therefore retained within the cell longer than non-residualizing radiolabels [35]. The KS83 minibody was conjugated to DOTA and binding of the immunoconjugate to EMP2 was evaluated by flow cytometry (Fig. 4a, b). The conjugation conditions were investigated at different molar ratios of DOTA to KS83 minibody in order to maximally preserve the binding to EMP2 (Fig. 4a). The optimal ratio of DOTA to KS83 minibody was found to be 20:1, and the number of DOTA per minibody (~3) was estimated based on the 20 lysines residues present in the KS83 minibody. At this conjugation ratio, binding activity was similar to that of the unconjugated minibody (Fig. 4b). The EMP2-binding of the DOTA-conjugated KS83 minibody was verified by ELISA using EMP2 peptide-coated plates (Fig. 4c). Serial dilutions of unconjugated KS83 and DOTA-conjugated KS83 minibody revealed similar EC_50_ of 24 ng/ml (0.30 nM) and 29 ng/ml (0.36 nM), respectively. Hence, the 20:1 DOTA to minibody ratio was used in all subsequent conjugation reactions.

### Small-Animal PET Imaging using Anti-EMP2 ^64^Cu-DOTA-Conjugated KS83 Minibody

**Fig. 5:**
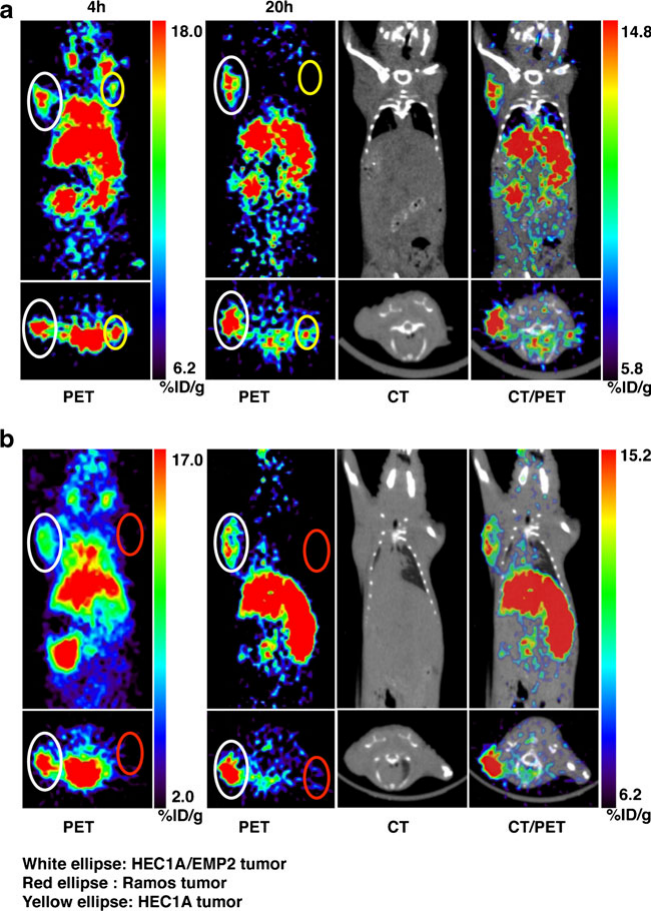
Small-animal PET imaging of EMP2 using ^64^Cu-DOTA-KS83 minibody. **a** Small-animal PET coronal images of mice bearing HEC1A/EMP2 (left shoulder, denoted *white ellipse*) and HEC1A (right shoulder, denoted *yellow ellipse*) tumor xenografts following administration of ^64^Cu-labeled anti-EMP2 DOTA-KS83 minibody at 4 and 20 h. *Bottom inset* shows a transverse image from the same animal. **b** Small-animal PET coronal images of mice bearing xenografts from HEC1A/EMP2 (left shoulder, denoted *white ellipse*) and Ramos (right shoulder, denoted *red ellipse*) cells. PET, CT, and PET/CT overlay images are shown at 20 h time point. *Bottom inset* depicts a transverse image from the same animal. Images at both time points are representative of five or more mice.

The DOTA-KS83 minibody was radiolabeled with ^64^Cu, and tumor targeting was evaluated in mice bearing HEC1A/EMP2, HEC1A, and Ramos (EMP2-negative) xenografts. Although both wild-type HEC1A and HEC1A/EMP2 cells were injected on the same day, the growth rates of these cells differ *in vivo* [8]. The average weight of HEC1a/EMP2 xenografts was 158 ± 50 while HEC1A wild-type tumors were significantly smaller (26 ± 11 mg). As a negative control, Ramos cells were injected and imaged when they reached 232 ± 23 mg. The radiolabeling efficiency for DOTA-KS83 minibody was 97 %, and the immunoreactivity, or the biologically active fraction is the 64Cu-DOTA-KS83 minibody that still retains binding to the target, was 84 %. Decay-corrected coronal micro-PET images were obtained at 4 and 20 h after intravenous injection with ^64^Cu-DOTA-KS83 minibody (Fig. 5a).

At both time points, HEC1A/EMP2 and wild-type tumors were clearly visualized, and a representative image at 20 h showed strong uptake in HEC1A/EMP2 tumor (white ellipse). As the HEC1A tumor is small, minimal uptake was visible in this coronal layer, but uptake was observed within the transverse profile (yellow ellipse) (Fig. 5a). In contrast, the EMP2-negative Ramos tumor had low minibody uptake (Fig. 5b). Radioactive uptake was also observed in the lung, heart, liver, and kidney at 4 h post-injection, with high activity persisting in the liver and kidney at 20 h post-injection.

Levels of radioactive uptake (%ID/g) at 4 and 20 h were quantitated by both ROI analysis and biodistribution studies (Table 2). At 4 h, ROI analysis showed significantly higher radioactive uptake using the ^64^Cu-DOTA-KS83 minibody in HEC1A/EMP2 tumors (7.6 ± 1.2 %ID/g) relative to HEC1A (5.6 ± 0.7 %ID/g; *P* < 0.05) and Ramos tumors (2.6 ± 1.0 %ID/g; *P* < 0.001). HEC1A tumors also displayed significantly more radioactive uptake compared with that of Ramos tumor (*P* < 0.01). RO1 analysis also detailed elevated radioactive uptakes in the kidneys, liver, heart, and lungs, but low levels were observed within soft tissue. After 20 h, HEC1A/EMP2 tumors showed a 1.7-fold increase in ^64^Cu-DOTA-KS83 minibody localization compared with the wild-type tumor (*p* < 0.01) and a 5.4-fold increase in localization compared with Ramos tumors (*P* < 0.001). The difference between HEC1A and Ramos tumors was also significant as wild-type tumors showed a 3.9-fold increase in radiolabeled KS83 localization (*P* < 0.05). In order to validate the ROI uptakes, tumors and the major organs were excised from mice at either 4 or 20 h post-injection, and the activity in the organs was counted in order to determine the percent injected dose per gram (Table 2). The counts from ROI analysis and *ex vivo* biodistribution were similar except that a slight difference was observed in the kidney.

**Table 2:** Estimated activities from drawn ROIs and uptakes measured by *ex vivo* weighing and counting derived from ^64^Cu-DOTA-KS83 (*n* = 5)

Organ (%ID/g)	ROI (4 h)	ROI (20 h)	Bio (4 h)	Bio (20 h)
HEC1A/EMP2 tumor	7.6 ± 1.2	10.2 ± 2.6	7.3 ± 0.5	9.7 ± 1.9
Hec1A tumor	5.6 ± 0.7	6.0 ± 0.1	5.8 ± 0.8	4.2 ± 1.3
Ramos tumor	2.6 ± 1.0	1.9 ± 0.5	N.D.	2.3 ± 0.5
Liver	19.6 ± 1.9	19.6 ± 3.5	19.2 ± 5.4	18.1 ± 1.6
Kidney	26.8 ± 4.6	24.8 ± 5.9	47.1 ± 12.4	36.4 ± 8.3
Lung	9.7 ± 1.6	4.8 ± 1.2	12.5 ± 5.6	5.5 ± 1.1
Heart	13.9 ± 2.8	4.2 ± 0.4	9.2 ± 0.9	4.3 ± 1.0
Soft tissue	0.7 ± 0.2	0.7 ± 0.2	1.0 ± 0.4	0.8 ± 0.1
Blood	N.D.	N.D.	18.1 ± 3.0	3.7 ± 0.6
Uterus	N.D.	N.D.	5.4 ± 0.8	4.9 ± 1.6

Data are expressed as %ID/g ± SD

*N.D.* not determined

The data generated suggest that anti-EMP2 minibodies localize specifically and with sensitivity to EMP2 positive tumors. However, an alternative possibility may be that EMP2 positive tumors differ in their vascularity and/or preferentially retain the radiometal labeled minibody. To explore the second possibility, experiments were performed using a ^64^Cu-DOTA CD20 minibody on HEC1A/EMP2 tumors. Table 3 compares the biodistribution of the control isotype tracer with the KS83 minibody at time of killing (4 and 20 h). Anti-EMP2 minibodies showed a 1.2- and 3-fold increase in uptake compared with radiolabeled anti-CD20 at 4 and 20 h, respectively.

**Table 3:** Biodistribution (bio) uptake of a control isotype tracer ^64^Cu-DOTA-CD20 at time of killing (*n* = 2)

Organ (%ID/g)	4 h	20 h
Hec1A/EMP2 tumor	6.1 ± 0.1	3.4 ± 0.6
Liver	19.0 ± 3.1	26.6 ± 0.9
Kidney	33.1 ± 8.6	24.2 ± 8.2
Lung	12.1 ± 2.6	6.2 ± 0.9
Heart	8.3 ± 2.1	6.2 ± 0.7
Soft tissue	1.1 ± 0.8	0.8 ± 0.3
Blood	13.2 ± 1.6	3.1 ± 1.9
Uterus	5.6 ± 2.7	4.9 ± 0.6

Mean is measured with SD

## Discussion

EMP2 is a tetraspan protein that is upregulated in a number of gynecological cancers, including endometrial and ovarian [3, 13]. Using a large panel of mouse and human biospecimens, we observed a restricted distribution of EMP2 protein expression in normal tissues in both species, suggesting the utility of EMP2 as a therapeutic and pharmacodiagnostic target. While EMP2 expression in human tissues has been previously studied, this has largely been evaluated at the level of messenger RNA (mRNA) rather than protein expression; however, the latter is most relevant to imaging strategies. In the present study, we show that EMP2 protein expression is largely concordant with mRNA expression, with EMP2 highly expressed in alveolar epithelium of the lung [28, 29], in the retinal pigmented epithelium in the eye [22], within secretory endometrium [23, 30], and in epithelia of the vagina and fallopian tube. In contrast, EMP2 was undetectable in many human tissues, including the small and large intestines, pancreas, liver, spleen, anovulatory endometrium, and kidney.

There is an unmet need for novel imaging strategies for clinical management of endometrial and ovarian cancers. In this paper, we show as a proof of principle that EMP2 expression can serve as a target for imaging. We have previously shown that the KS83 diabody recognizes both murine and human EMP2 and has therapeutic potential as seen in a number of tumor models [3, 13]. We thus hypothesized that imaging of EMP2 may serve as an early monitor of response to tailored therapy or be useful prior to surgical resection to identify metastatic disease. Therefore, we reformatted the variable genes of the KS83 diabody [13] into a minibody of approximately 80 kDa in size whose pharmacokinetics and biodistribution are particularly favorable compared to other antibody fragments for tumor imaging [21]. Minibodies have a slower clearance compared to the diabody largely due to their increased molecular weight, and they have been shown to be useful for PET imaging [20].

Preliminary experiments showed that antibody fragment binding to EMP2 caused the target to internalize. Indeed, initial imaging studies using nonresidualizing anti-EMP2 antibody fragments (prepared with I-124 by the Iodogen method) produced poor retention in the EMP2-positive tumor xenografts (data not shown). This was not unexpected as antigen internalization releases radioiodine by dehalogenases either directly or after the protein is degraded to iodotyrosines, resulting in loss of signal and low percent injected dose per gram [36].

Accordingly, in this study, we prepared a residualizing anti-EMP2 radiolabel using the bifunctional chelating agent DOTA to conjugate minibody with a chelator for ^64^Cu [24, 37–39]. ^64^Cu-DOTA has been shown to have moderate stability *in vivo*, which leads to a high hepatic radioactivity uptake [40]. ^64^Cu-radiolabeled complexes with increased stability have been studied with 1,4,8,11-tetraazacyclotetradecane-*N*,*N*′,*N*″,*N*′″-tetraacetic acid [40, 41], but this chelator requires harsh reaction conditions such as elevated temperature for complexation with the radiometal. Although the 1,4,7-triazacyclononane-*N*,*N*′*,N*″-triacetic acid can be labeled in mild conditions and provides better stability of Cu-chelate complexes [42, 43], it has not been evaluated in the clinic. Thus, in this study, we used DOTA-mono-*N*-hydroxysuccinimide ester (DOTA-NHS-ester) conjugated with lysine residues of KS83 minibody in order to radiometal label with ^64^Cu. Since lysine residues are present in loops forming the antigen binding site, modification of the lysine residues could potentially affect binding to the antigen. Indeed, molar ratios of 50:1 and over greatly impacted binding to EMP2 (Fig. 4a). However, a molar ratio of 20:1 largely preserved the antigen binding activity compared to unmodified KS83 minibody. Hence, this conjugation strategy proved to be suitable for radiolabeling the anti-EMP2 KS83 minibody.

Non-invasive serial PET imaging was used to generate data in the same animal, providing important information of how the compound moves through the body over time. Indeed, PET images of tumor bearing mice injected with ^64^Cu-DOTA-KS83 minibody produced excellent radioactive uptake in EMP2-positive tumors. At 20 h, which is the optimal time for minibody imaging [37], drawn ROIs revealed 10.2 ± 2.6 %ID/g ± SD in the HEC1A/EMP2 tumor, a 5.4-fold uptake ratio compared to that in the EMP2-negative tumor (1.9 ± 0.5). These findings were confirmed directly *ex vivo* by measuring the radioactivity in excised organs.

In order to determine the sensitivity of ^64^Cu-DOTA-KS83 minibody to EMP2, xenografts were created using both wild-type HEC1A and HEC1A/EMP2 cells. Published reports have shown that there is a 2- to 4-fold increase in EMP2 expression by Western blot analysis [8]. Imaging with the ^64^Cu-DOTA-KS83 minibody demonstrated targeting to both HEC1A/EMP2 (10.2 ± 2.6 % ID/g) and HEC1A (6.0 ± 0.1 % ID/g) tumors, with HEC1A/EMP2 exhibiting a 1.7-fold higher level of radioactivity compared to HEC1A (*P* < 0.01). Moreover, the high ratio of EMP2-positive tumor relative to various normal tissues lacking EMP2 protein expression (e.g., soft tissue, heart, blood) suggests that EMP2 minibodies specifically and efficiently adhere to their antigen. To verify that the retention was not due to permeability and retention effects, HEC1A/EMP2 xenografts were targeted using a control isotype tracer. Injections with radiometal-labeled anti-CD20 minibody showed low levels of tracer within the tumor, suggesting that the high radio-uptake of labeled EMP2 minibody was specific.

Of note, high radioactive uptake of ^64^Cu-DOTA-KS83 minibody was observed in liver and kidney even though these are not sites of EMP2 expression. This uptake may be attributed in part to the minibody or the radiometal itself. The liver uptake was expected, due to the size of the minibody that is above the renal threshold for clearance (>60 kDa) and possibly by avid transchelation of the radiometal ^64^Cu [40]. The elevated renal uptake was unexpected, but may reflect the small fraction of partially metabolized ^64^Cu-DOTA-KS83 minibody, which despite residualizing properties are released into circulation from primary tissue binding sites and reach the kidney where the partially metabolized products may be retained. This may also explain the slightly elevated levels of agent detectable at 20 h in the blood and heart (the latter largely reflecting a blood compartment signal). Consistent with this explanation, high radioactive uptake was also observed using the ^64^Cu-DOTA-CD20 minibody.

The present and previous studies show that EMP2 is highly expressed in normal lung (type 1 pneumocytes) [44]. As the minibody recognizes both human and murine EMP2, it is notable that lung uptake of ^64^Cu-DOTA-KS83 minibody is <2-fold higher than the activity in the blood. This suggests that EMP2 in native pulmonary epithelium may be relatively sequestered from access to ^64^Cu-DOTA-KS83 minibody. Consistent with this hypothesis, localization of EMP2 in polarized epithelia is restricted to zona adherens (Morales and Gordon, unpublished data). Nonetheless, the expression of EMP2 may make it difficult to detect and characterize lung metastases. Additional experiments will be needed to verify these and other utilities.

In conclusion, EMP2 is novel cancer biomarker, notably for endometrial and ovarian cancer, that has been previously shown to be an independent prognostic indicator, and an effective target in preclinical recombinant antibody therapy. In this study, we characterize a residualizing imaging agent, a ^64^Cu-labeled minibody to EMP2, and demonstrate its effectiveness in imaging human EMP2-positive endometrial tumor xenografts. We predict that with further refinement anti-EMP2 antibody fragments may be have value for imaging of tumor localization and therapeutic response in patients with EMP2-positive malignancies.
